# RNA-Seq Based Toxicity Analysis of Mesoporous Polydopamine Nanoparticles in Mice Following Different Exposure Routes

**DOI:** 10.3389/fbioe.2022.893608

**Published:** 2022-04-28

**Authors:** Zihua Huang, Luoyijun Xie, Jifan Zhang, Qiyan Li, Yulin Liu, Xuemei Fu, Miaomiao Yuan, Qingjiao Li

**Affiliations:** The Eighth Affiliated Hospital, Sun Yat-sen University, Shenzhen, China

**Keywords:** transcriptome sequencing, MPDA NPs, intravenous injection, intramuscular injection, intragastric administration

## Abstract

Mesoporous polydopamine nanoparticles (MPDA NPs) are promising nanomaterials that have the prospect of clinical application for multi-strategy antitumor therapy, while the biosecurity of MPDA NPs remains indistinct. Here, transcriptome sequencing (RNA-Seq) was performed to systematically reveal the toxicity of MPDA NPs to five categories of organs after three different exposure routes, including intravenous injection, intramuscular injection, and intragastric administration. Our results uncovered that MPDA NPs could be deposited in various organs in small amounts after intravenous administration, not for the other two exposure routes. The number of differentially expressed genes (DEGs) identified in the heart, liver, spleen, lung, and kidney from the intragastric administration group was from 22 to 519. Similarly, the corresponding number was from 23 to 64 for the intramuscular injection group and was from 11 to 153 for the intravenous injection group. Functional enrichment analyses showed 6, 39, and 4 GO terms enriched for DEGs in intragastric administration, intramuscular injection, and intravenous injection groups, respectively. One enriched pathway was revealed in intragastric administration group, while no enriched pathway was found in other groups. Our results indicated that MPDA NPs produced only slight changes at the transcriptome level in mice, which provided new insights for further clinical application of MPDA NPs.

## 1 Introduction

An increasing number of nanotechnologies are being developed for medical applications, including drug and gene delivery, clinical diagnostics as well as improved imaging agents, and several nanoparticle-based delivery systems have entered clinical trials (English and Aloi, 2015; Verma et al., 2015; Stidl et al., 2016). In parallel, the potential harm to public health and the environment brought by nanotechnologies has caught the attention of researchers. In the past decade, the biosafety of nanomaterials has been one of the major obstacles to the further use of nanoparticles in medical applications (Greish et al., 2018).

It was reported that polydopamine (PDA) had been widely utilized in tumor nanodrug delivery systems (Wang et al., 2018; Zeng et al., 2018; Li et al., 2020), which could significantly improve the drug distribution in the body, enhance the curative benefits as well as reduce toxic and side effects. However, traditional PDA nanoparticles have a finite specific surface area, and the loaded drugs are easily separated under complex physiological conditions, limiting the drug-loading efficiency and tumor suppression efficiency. To solve these problems, Tang et al. (2015) synthesized mesoporous polydopamine nanoparticles (MPDA NPs) based on a preparation procedure for mesoporous carbon material using high-molecular-weight block copolymers as templates (Tang et al., 2015), which provided new insight into high drug-loading capacity, multimodal anticancer treatment, and visual therapy of nanoparticles. Despite the fascinating applicability of MDPA NPs, the toxicity and safety of MDPA NPs remained unknown, which prevented further clinical application.

The exposure route of the nanomaterials is a key factor in the interaction between nanomaterials and the human body. More specifically, various exposure routes could affect the toxicity, distribution, metabolism as well as their performance as nanomedicine of nanomaterials in the human body. For example, Elbrink et al. (2021) evaluated the impact of drug loading and the administration routes for solid lipid nanoparticles (SLNs) and revealed that the subcutaneous injection performed better than the intramuscular injection and intravenous administration owing to lower blood perfusion in the subcutaneous tissues (Elbrink et al., 2021). In addition, Yu et al. (2017) systematically studied the distribution of polyethyleneimine-modified NaYF4:Yb, Er upconversion nanoparticles (PEI@UCNPs) in mice under different exposure routes and observed that a large number of PEI@UCNPs were deposited in the spleen within 30 days in the intraperitoneal administration group while PEI@UCNPs *via* intragastric administration exhibited an accumulation that decreased with time in various body tissues (Yu et al., 2017). Hence, it was necessary to systematically assess the biosafety of MPAD NPs undergoing various administration routes, which was one of the important steps before clinical application.

To understand and predict the toxicity of a compound at a systems level, global transcriptome sequencing (RNA-Seq) is undoubtedly the most optimal approach. For instance, Yang et al. (2020) revealed the modulation of gene expression in the liver and lungs after treatment with ZnO quantum dots (QDs) by using RNA-Seq, and the changed transcripts were used to infer the potential toxicity of ZnO QDs (Yang et al., 2020). On the one hand, RNA-Seq detects unknown transcripts, while the DNA microarrays could only detect specific transcripts since the probes are designed with specific nucleotide sequences (Kaliyappan et al., 2012; Casamassimi et al., 2017). On the other hand, RNA-Seq enables the quantification of gene expression levels and allele-specific expression in a single experiment, as well as the identification of novel genes, splice isoforms, and fusion transcripts (Casamassimi et al., 2017).

In this study, we systematically examined the toxicity of MPDA NPs with three different exposure routes, including intravenous injection, intramuscular injection, and intragastric administration. After injection of MPDA NPs to female mice for 7 days, histopathology observation was performed to measure the degree of deposition of MPDA NPs and morphological changes at the cell level on five categories of tissues including the heart, liver, lung, kidney, and spleen. Next, with the application of RNA-Seq technology, differentially expressed genes (DEGs) were acquired between the MPDA NP injection group and the control group. At last, the gene ontology (GO) and Kyoto encyclopedia of genes and genomes (KEGG) pathways annotation of DEGs were used to access the possible toxicity of the MPDA NPs at the transcriptome level. This study will be helpful for exploring the possibility of MPDA NPs in further clinical application, and especially for selecting the appropriate exposure routes.

## 2 Material and Methods

### Materials

Dopamine hydrochloride and 1,3,5-trimethylbenzene (TMB) were purchased from Aladdin Reagent (Shanghai, China). Ammonia aqueous solution (NH_3_·H_2_O, 30 wt%) was purchased from Macklin (Shanghai, China). Pluronic F127 was purchased from Sigma-Aldrich (MO, United States). Phosphate buffered saline (PBS) was bought from Gibco (Shanghai, China). Anhydrous ethanol was bought from Aladdin Reagent (Shanghai, China). Chloral hydrate was bought from Xiya (Chengdu, China). Dopamine hydrochloride was bought from Aladdin Reagent (Shanghai, China).

### Synthesis and Characterization of MPDA NPs

MPDA NPs were synthesized according to the procedure which had been previously reported (Chen et al., 2016). The structural and elemental distribution of MPDA NPs were detected by transmission electron microscope (TEM). The morphology of MPDA NPs was measured by scanning electron microscope (SEM). The particle size and zeta potential of MPDA NPs were determined by dynamic light scattering (DLS). The crystal structure was analyzed by X-ray powder diffraction (XRD). The surface area and pore diameter were determined by Brunauer–Emmett–Teller (BET).

### Animals and Experiments

Twelve BALB/c mice (female, aged 4 weeks old, weight 20 ± 2 g) were supplied by the Guangdong Medical Laboratory Animal Center (Guangdong, China). To get mice to acclimate to the new environment, they were fed in rearing rooms for a week before intervention. The temperature in the rearing room was controlled at 20 ± 3°C and the relative humidity was from 30 to 73%. The animals were housed (3/cage) and they were allowed to walk around the cage, eat and drink freely. The animal studies were approved by IACUC of The Eighth Affiliated Hospital, Sun Yat-sen University (2022-002-01).

After 1-week acclimation, 12 female BALB/c mice were randomly divided into 4 groups of 3 mice each (Table 1), and the mice of experimental groups were accepted MPDA NPs treatment for one time. In the following 7 days, the mice were allowed to walk around the cage, eat and drink freely, and their weight was recorded every day. On day 7, all mice were taken under anesthesia for cervical dislocation, and then organs were weighted and collected, including the heart, liver, spleen, kidney, and lung.

**TABLE 1:** Basic administration information of the mice exposed by the intragastric (i.g.) administration, intramuscular (i.m.) injection, and intravenous (i.v.) injection.

Group	Dose (mg)	Day	The number of mice
Control	0	7	3
i.g.	50	7	3
i.m.	40	7	3
i.v.	8	7	3

### Organ Indexes

After the dissection of mice, the fat and fascia adhered to the exposed organs were carefully removed with surgical forceps, and then the organs were subsequently placed in cold PBS to wash away residual blood. After carefully drying, the organs were weighed immediately with an electronic balance. The following formula was used to calculate the organ indexes.

organ indexes(%)=(W1 )/W2∗100%

where W_1_ is the weight of the organ and W_2_ is the weight of the corresponding mouse.

### Histopathological Examinations

After weighting, the needed tissues were immediately fixed in a 10% formalin solution. The fixed tissues were embedded in paraffin blocks and then sectioned into 5 mm sections and mounted on the slides. The slides were observed after hematoxylin and eosin staining (HE). Using a light microscope, the images were observed at a magnification of 100X.

### RNA Extraction, cDNA Library Construction, and Sequencing

#### RNA Extraction

The TRIzol reagent (Invitrogen, Carlsbad, CA, United States) was used to extract total RNA from tissues of mice. First, RNA degradation and contamination were detected by agarose gel electrophoresis. The purity and concentration of RNA were assessed with Nanodrop. Second, to get more accurate information, the concentration and integrity of RNA were measured with a Qubit 2.0 Fluorometer (Life Technologies, Carlsbad, CA, United States) and an Agilent 2100 bioanalyzer (Santa Clara, CA, United States), respectively.

#### cDNA Library Construction

After RNA extraction, sequencing libraries were generated using the NEBNext® UltraTM RNA Library Prep Kit for Illumina® (NEB, United States), and each sample was labeled with an index code at the beginning.

First, mRNA was separated from total RNA using poly-T oligo-attached magnetic beads. Next, using divalent cations under elevated temperature, mRNA was fragmented in NEBNext First Strand Synthesis Reaction Buffer (5X). Then, the first-strand of cDNA was synthesized using a random hexamer primer and M-MuLV Reverse Transcriptase (RNase H). The second-strand cDNA was subsequently synthesized using DNA Polymerase I and RNase H. The remaining overhangs were converted into blunt ends via exonuclease/polymerase activities. After adenylation of 3′ ends of DNA fragments, NEBNext Adaptor with hairpin loop structures were ligated to prepare for hybridization. Then, the library fragments were purified with the AMPure XP system (Beckman Coulter, Beverly, United States) to select cDNA fragments of specified length interval 250–300 bp. Next, 3 μl USER Enzyme (NEB, United States) was used with size-selected, adaptor-ligated cDNA at 37°C for 15 min, followed by 5 min at 95°C before PCR. Then PCR was performed with Phusion High-Fidelity DNA polymerase, Universal PCR primers, and Index (X) Primer. Finally, PCR products were purified (AMPure XP system), and library quality was assessed on the Agilent Bioanalyzer 2100 system.

#### Sequencing

The library preparations were sequenced on an Illumina Novaseq 6000 platform (Novogene, Beijing, China) and 150 bp paired-end reads were generated.

### Trimming, Transcriptomic Assembly, and Gene Annotation

The raw sequencing data obtained from the Illumina platform contained sequence artifacts, including reads containing adapter contamination, low-quality nucleotides, and unrecognizable nucleotide (N), which would cause errors in the following data analyses steps. Therefore, the downstream analysis was based on clean data which was transformed from raw data using Fastp software (Chen et al., 2018) according to the following standard: 1) Discarding a paired-end reads if either one end contains adapter contamination; 2) discarding a paired-end reads if more than 10% of bases are uncertain in either one end; 3) discarding a paired-end reads if the proportion of low quality (Phred quality <5) bases is over 50% in either one end. Next, the paired-end were mapped to the mouse genome [mm10, Genome Reference Consortium Mouse Build 38 (GCA_000001635.2)] using HISAT2 software (Kim et al., 2019). Counts for each gene were obtained using HTSeq software (Anders et al., 2015). Sequencing statistics for each sample were summarized in Supplementary Table S1.

### Analyses of Differentially Expressed Genes

The differentially expressed genes between each treated group and the control group (*n* = 3 per group) were determined by the DESeq2 R package (Love et al., 2014), which is a popular method for differential analysis of count data. The DEGs were determined based on two criteria: 1) |log2 (fold change) | > 1 and 2) adjust *p* value <0.05. FPKM (fragments per kb per million reads) were transformed from count data in R (https://www.r-project.org/). To visualize the overall distribution of the DEGs, the volcano plots based on count data and the heatmaps based on FPKM data were created in R.

### Functional Annotation of DEGs

To determine the functional annotation, GO enrichment (Harris et al., 2004), and KEGG pathway (Kanehisa and Goto, 2000), enrichment analyses were performed for the DEGs using the clusterProfiler R package (Yu et al., 2012). Moreover, three categories were included in enriched GO terms, which are biological processes (BP), cellular components (CC), and molecular functions (MF). The enriched GO terms were selected based on two standards: 1) adjust *p* value <0.05 and 2) the number of unique genes in each GO term was more than one. Similarly, the enriched KEGG pathways were selected based on two standards: 1) adjust *p* value <0.05 and 2) the number of unique genes in each KEGG pathway was more than one.

## 3 Results

### Characterization of MPDA

**FIGURE 1:**
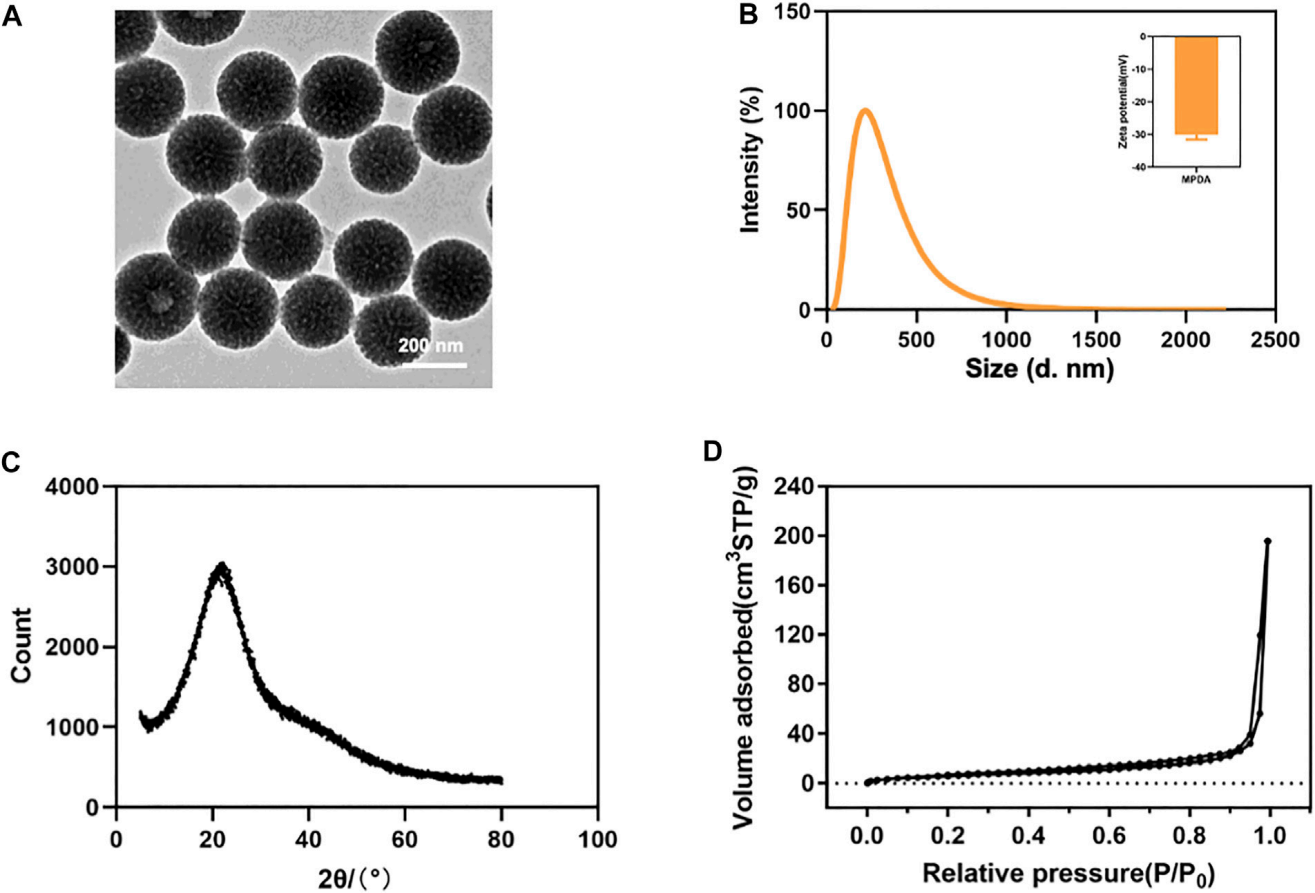
**(A)** TEM image of MPDA NPs. **(B)** Size distribution and zeta potentials of MPDA NPs. **(C)** XRD spectrum of MPDA NPs. **(D)** N2 adsorption-desorption isotherms of MPDA NPs.

Both TEM and SEM observations displayed spherical morphology and mesoporous structure with a clear arrangement and uniform size distribution of MPDA NPs (Figure 1A, Supplementary Figures S1A, B, C). As shown in Figure 1B, the DLS measurements showed that the average hydrated particle size was roughly 223 nm, and the zeta potential was approximately -30 mV, indicating that the MPDA NPs surface had a negative charge. At the same time, the element mapping of MPDA NPs showed that C, N, and O elements were evenly distributed throughout the nanoparticles, with the C skeleton forming the core structure of MPDA NPs (Supplementary Figure S2), which was consistent with the XRD results. As shown in Figure 1C, the XRD spectrum of MPDA NPs exhibited a wide peak centered at approximately 2θ = 22°, and the corresponding crystal plane was (002), which verified that the main composition of MPDA was carbon element (Chen et al., 2016; Peng et al., 2019; Wang et al., 2019). The broad peak also indicated that MPDA NPs did not belong to crystalline materials (Ili Balqis et al., 2017). In addition, the MPDA NPs showed a BET surface area of 28.882 m^2^ g^−1^ (Figure 1D), which enabled them to load the drug efficiently. According to the BJH model, the pore diameter of MPDA NPs was about 1.75 nm and its pore volume was 0.311 cm^3^ g^−1^ (Supplementary Figure S1D).

### Mortality, Body Weight, and Organ Indexes of Mice

In intravenous and intramuscular injection groups, mice were administrated with MPDA NPs suspended in PBS at 40 and 8 mg for a single time, and in the intragastric administration group, mice were administrated with MPDA NPs suspension at 50 mg at two points in time, 6 hours had passed between them. It was noticeable that the maximum tolerated dose (MTD) of intragastric administration was higher than that of other exposure routes. During the 7-day period, no mouse deaths were observed. Furthermore, no abnormal behaviors including vocalizations, labored breathing, difficulties moving, hunching or unusual interactions with cage mates were observed as well.

**FIGURE 2:**
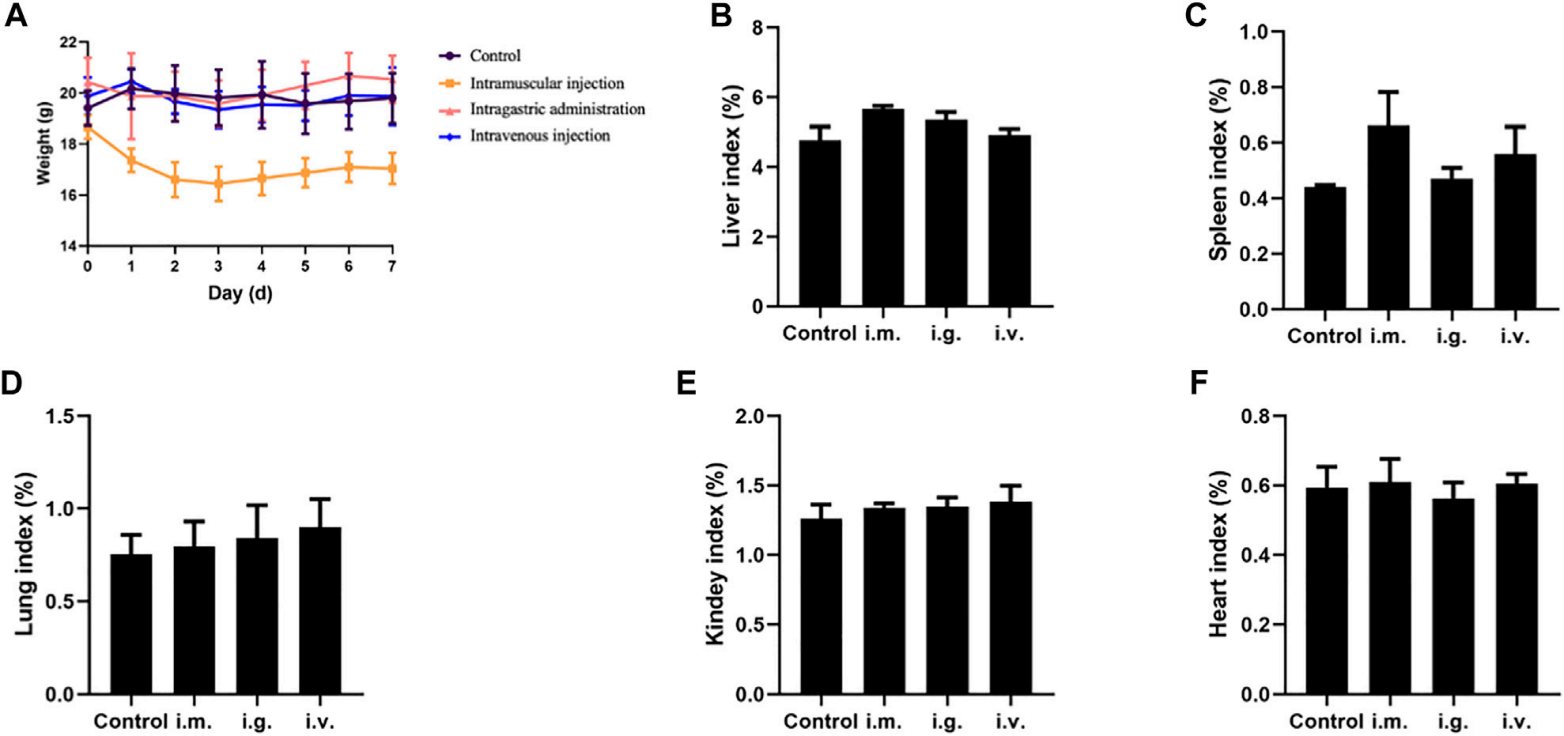
**(A)** Body weight curve of mice. **(B–F)** Organ indexes of tissues from the mice exposed by intramuscular (i.m.) injection, intragastric (i.g.) administration, and intravenous (i.v.) injection, for the liver, spleen, lung, kidneys, and heart, respectively.

As shown in Figure 2A, the weight of mice in each group was relatively stable, but the weight of mice in the intramuscular injection group saw a slight decrease in the first 3 days. Organ indexes analysis was performed on the hearts, liver, spleen, lung, and kidneys of mice, and it was found that organ indexes did not alter significantly after different exposure routes to MPDA NPs (Figures 2B–F).

### Histopathological Examinations

**FIGURE 3:**
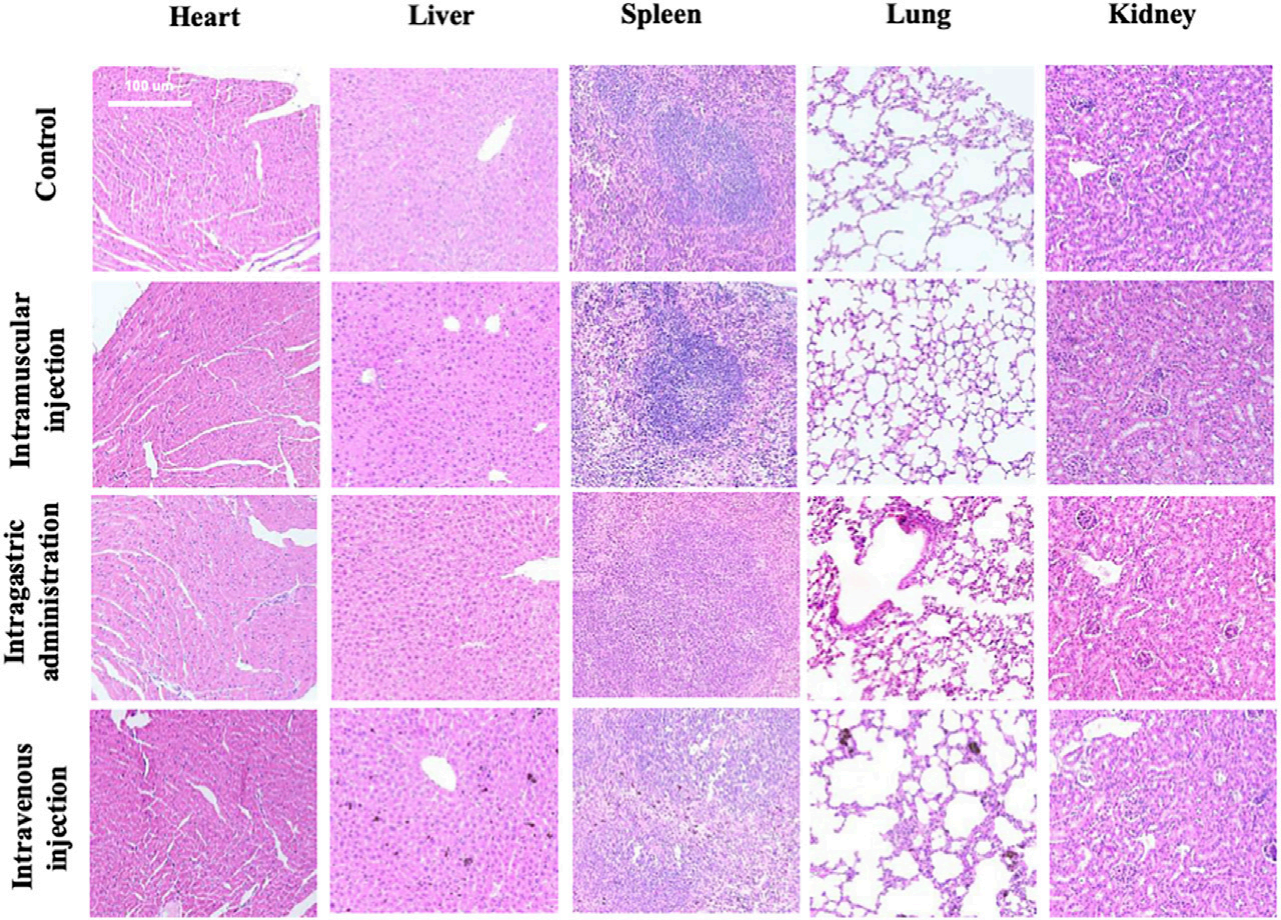
Histological examination of tissues (heart, liver, spleen, lung, and kidneys) from the mice exposed by intramuscular (i.m.) injection, intragastric (i.g.) administration, and intravenous (i.v.) injection at day 7. The scale bar is 100 μm.

MPDA NPs did not induce any changes in both the shape and volume of the organs on day 7 by different exposure routes, while the color of the spleen and liver in the intramuscular injection group and spleen, liver, and lung in the intravenous injection group became darker than the control group (Supplementary Figure S3). Furthermore, Figure 3 depicted the micromorphology of the organs of mice in each group after exposure to MPDA NPs. It could be observed that a small amount of MPDA NPs deposited in the liver, spleen, and lungs in the intravenous injection group, while no nanoparticles were observed in the organs of mice in the other two treated groups. It meant that some MPDA NPs could deposit in tissues passing through biological barriers and could not be excreted within 7 days. In addition, compared to the control group, the organs of mice in each experimental group did not display any histopathological changes.

### Analysis of Differentially Expressed Genes

**FIGURE 4:**
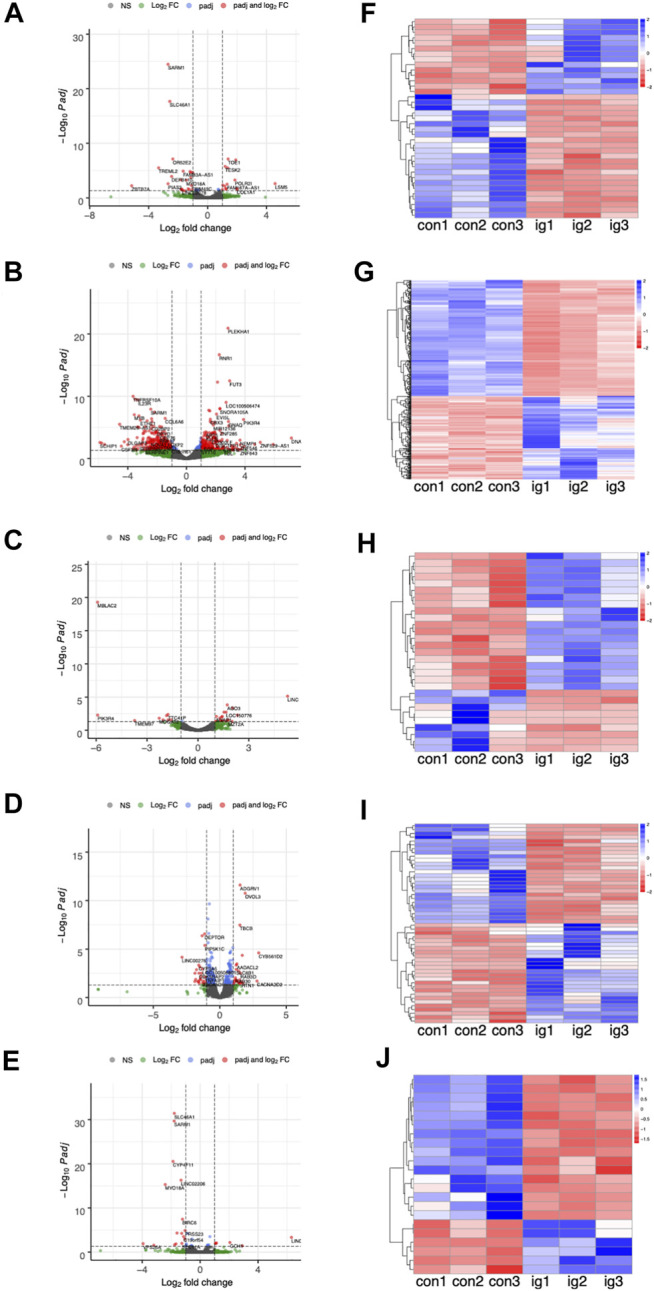
**(A–E)** The volcano plots of DEGs between the intragastric (i.g.) administration group and control group, for the heart, liver, spleen, lung, and kidneys, respectively. **(F–J)** The heatmap of DEGs.

The number of DEGs between the treated groups and control group were list in Table 2 and Supplementary Table S2. Furthermore, Figure 4 display the heatmaps and volcano plots of DEGs between the intragastric administration group and control group, for the five tissues, including heart, liver, spleen, lung and kidney separately. Also, the DEGs of intramuscular group and intravenous group were shown in Supplementary Figure S4 and Supplementary Figure S5. For the intragastric administration group, the organ with the largest number of DEGs was liver, up to 519. The rest of the organs had close numbers of DEGs, which were 37, 29, 52 and 22 identified in heart, spleen, lung and kidney separately. For the intramuscular injection group, the organ with the least number of DEGs was lung, down to 23. And 58, 64, 32 and 34 DEGs were identified in heart, liver, spleen, and kidney separately. For intravenous group, the organ with the least number of DEGs was lung, down to 11. And 124, 52, 153 and 93 DEGs were identified in heart, liver, spleen and kidney respectively. Furthermore, the number of up-regulated and down-regulated DEGs were close for each comparison (Supplementary Tables S3–S5).

**TABLE 2:** The number of DEGs of tissues (heart, liver, spleen, lung, and kidneys) from the mice exposed by the intragastric (i.g.) administration, intramuscular (i.m.) injection, and intravenous (i.v.) injection. (|log2 (fold change) | > 1, adjust *p* value <0.05).

Tissue	The number of DEGs		
	i.g.	i.m.	i.v.
Heart	37	58	124
Liver	519	64	52
Spleen	29	32	153
Lung	52	23	11
Kidney	22	34	93

### Functional Annotation of DEGs

**FIGURE 5:**
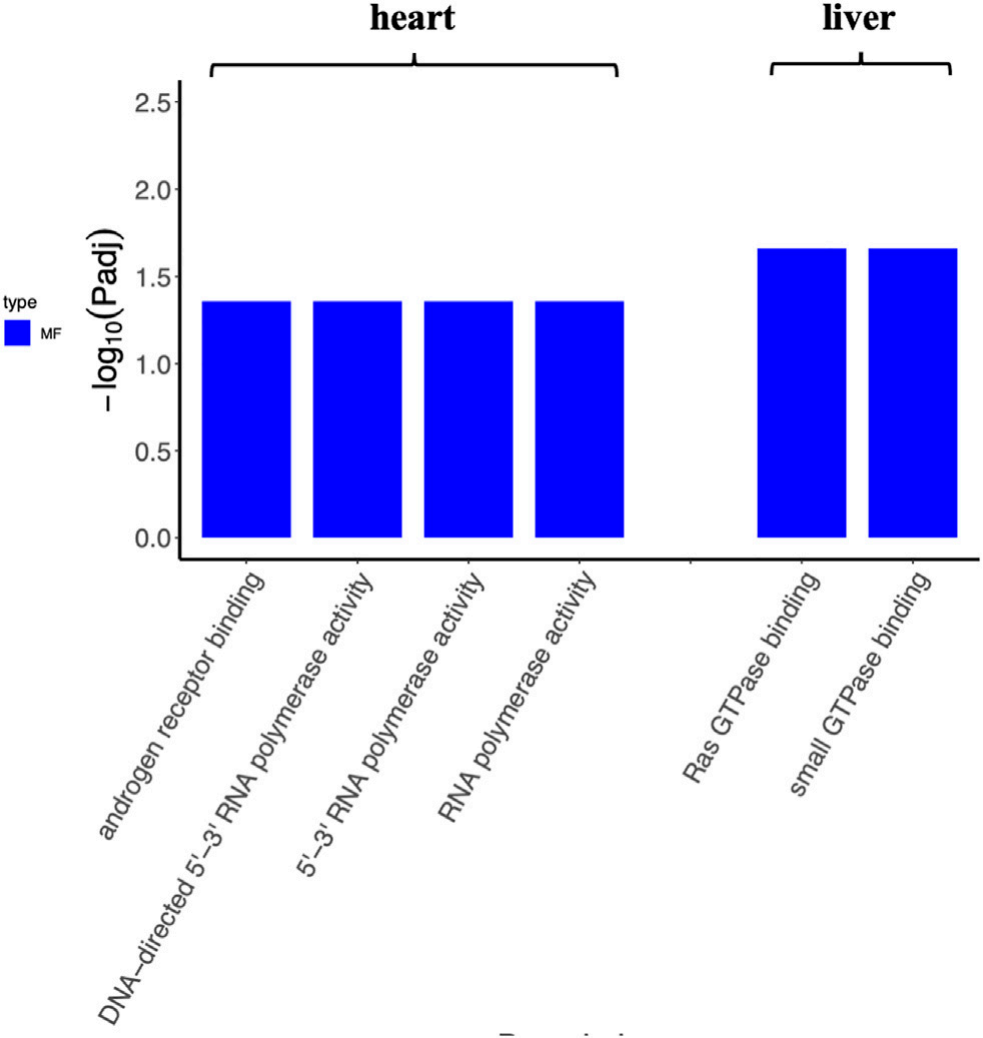
The enriched GO terms of tissues (heart and liver) from the intragastric administration group. No GO term is enriched for the spleen, lung, and kidney.

To determine the function of DEGs and metabolic pathway enrichment, GO (Table 3) and KEGG pathway analyses (Table 4, Table 5) were performed on DEGs in each group. For intragastric administration group, we obtained 6 enriched GO terms, including regulation of cellular response to androgen receptor binding (GO:0050681), polymerase activity (GO:0003899, GO:0034062, GO:0097747), and GTPase binding (GO:0017016, GO:0031267) (Figure 5, Supplementary Table S6). Only one enriched KEGG pathway was observed, which is calcium signaling pathway (mmu04020). For intramuscular injection group, we obtained 39 enriched GO terms, including transmembrane transporter activity (GO:0008509, GO:0046943, GO:0051184, GO:0046873, GO:0072349, GO:0005342, GO:0022803, GO:0015081), channel activity (GO:0015267, GO:0022836, GO:0005217, GO:0005216, GO:0022839, GO:0099094, GO:0022834, GO:0015276, GO:0022838) and so on (Supplementary Figure S6, Supplementary Table S7). No enriched KEGG pathway was observed in this group. For intravenous injection group, we obtained 4 enriched GO terms, including catenin complex (GO:0016342), apical junction complex (GO:0043296), mismatch repair complex binding (GO:0032404), and catalytic activity, acting on RNA (GO:0140098) (Supplementary Figure S7, Supplementary Table S8). No enriched KEGG pathway was found in this group as well.

**TABLE 3:** The number of enriched GO terms of tissues (heart, liver, spleen, lung, and kidneys) from the mice exposed by the intragastric (i.g.) administration, intramuscular (i.m.) injection, and intravenous (i.v.) injection. (adjust *p* value <0.05, # of unique gene >1).

Tissue	i.g.			i.m.			i.v.		
	BP	MF	CC	BP	MF	CC	BP	MF	CC
Heart	0	4	0	0	0	6	0	0	2
Liver	0	2	0	0	6	0	0	1	0
Spleen	0	0	0	0	21	4	0	0	0
Lung	0	0	0	0	0	0	0	1	0
Kidney	0	0	0	0	2	0	0	0	0

**TABLE 4:** The number of KEGG pathway of tissues (heart, liver, spleen, lung, and kidneys) from the mice exposed by the intragastric (i.g.) administration, intramuscular (i.m.) injection, and intravenous (i.v.) injection. (adjust *p* value <0.05, # of unique gene >1).

Group	The number of KEGG pathway		
	i.g.	i.m.	i.v.
Heart	0	0	0
Liver	0	0	0
Spleen	0	0	0
Lung	0	0	0
Kidney	1	0	0

**TABLE 5:** Information of the enriched KEGG pathway.

Group	Tissue	KEGG id	Description	Gene no.	Genes	p.adj
i.g.	Kidney	mmu04020	Calcium signaling pathway	2	Itpkc/Mcoln2	0.0496

## 4 Discussion

In the past few years, researchers mainly focused on the application of MPDA NPs, especially its application in tumor treatment. For example, Zhang et al. (2019) discovered an MPDA-based synthetic for chemo-photothermal therapy, polyethylene glycol-modified MPDA (PEG-MPDA), which had not only photothermal conversion efficiency but also high paclitaxel (PTX) loading content (Zhang et al., 2019). According to their result, the tumor ablation could be observed after MPDA-PEG-PTX treatment combined with 2 Wcm-2 laser irradiations for 5 min. In addition to chemo-photothermal therapy, MPDA could also be applied to immuno-photothermal, a combination of photothermal therapy and photodynamic therapy/chemodynamic therapy, as well as cancer theranostics (Hu et al., 2019; Huang et al., 2021; Wu et al., 2021). However, few studies have illustrated the potential toxicity of MPDA so far, which blocked further clinical application. In this study, we adopted RNA sequencing-based strategy to discover the toxicity of MPDA NPs.

In our pre-experiment, we exposed mice to MPDA for different durations (7 and 30 days) and then stained the heart, liver, spleen, lung, and kidney with HE staining. Figure 3 displays the tissues examined at day 7, while Supplementary Figure S8 (Supplementary Figure S8) displays the tissues examined on day 30. We did not observe any changes in the tissues with the different time periods of administration. Therefore, we decided to examine the tissues on day 7. As mentioned above, on day 7, the mice from the intramuscular injection group weighed approximately 2 g less than they did on day 1 while the weight of mice from the other two experimental groups did not significantly change. We assumed that the abnormal phenomenon was likely caused by the injection of a large volume of suspension rather than the toxicity of MPDA NPs. It was necessary to point out that the volume of MPDA suspension for this group was up to 1.6 ml, which was a lot for mice around 20 g. However, it was difficult to reduce the volume of suspension because the solubility of MPDA NPs was limited. Furthermore, we observed some MDPA NPs deposition in several tissues in intravenous group, which indicated the material has not been completely metabolized. In the intravenous injection group, deposited MPDA NPs were not observed in the heart and kidney, but could be observed in the liver, spleen, and lung of the same mouse. This phenomenon supported the different metabolic capacities of different visceral tissues to MPDA. The photographs we presented above just showed the result on day 7 and it might take more time to metabolize.

No enriched KEGG pathway was found in both intramuscular injection and intravenous injection groups. In parallel, only one KEGG pathway was identified in kidneys in the intragastric administration group, which was the calcium signaling pathway. Calcium ions are abundant in the human body, regulating significant physiological activities as an important signaling molecule. It is reported that the concentration of Ca^2+^ plays an important role in the regulation of nervous system excitability, the contraction of muscles, intestinal microbial activity, the activity of enzymes, and the biological clock (Simpson et al., 1995; Kon and Fukada, 2015). As shown in Table 5, two down-regulated genes were included in this pathway, suggesting the calcium signaling pathway might be inhibited after MPDA NPs intragastric administration. In other words, calcium concentration in the human body should be of concern when receiving MPDA treatment.

In conclusion, we investigated the toxicity of MPDA NPs on global gene expression of important organs by RNA-Seq. The DEGs detected in each organ were quite a few and only several GO terms, and one KEGG pathway were enriched for these DEGs. The results suggested that MPDA NPs did not cause great or serious changes in mice from three different administration routes, which shed light on the toxicity of MPDA NPs at the molecular mechanism level.

## Data Availability

The raw RNA sequencing data are available from Genome Sequence Archive (GSA) at The National Genomics Data Center (https://ngdc.cncb.ac.cn), with the accession number PRJCA008032.
