# Supplementary material for: RNA-Seq Based Toxicity Analysis of Mesoporous Polydopamine Nanoparticles in Mice Following Different Exposure Routes
Source: Front Bioeng Biotechnol. 2022 Apr 28;10:893608. doi: 10.3389/fbioe.2022.893608 (PMC9096556; doi:10.3389/fbioe.2022.893608)
Supplement: Supplementary file 1 [file DataSheet1.docx]

Supplementary Material

# Supplementary Figures


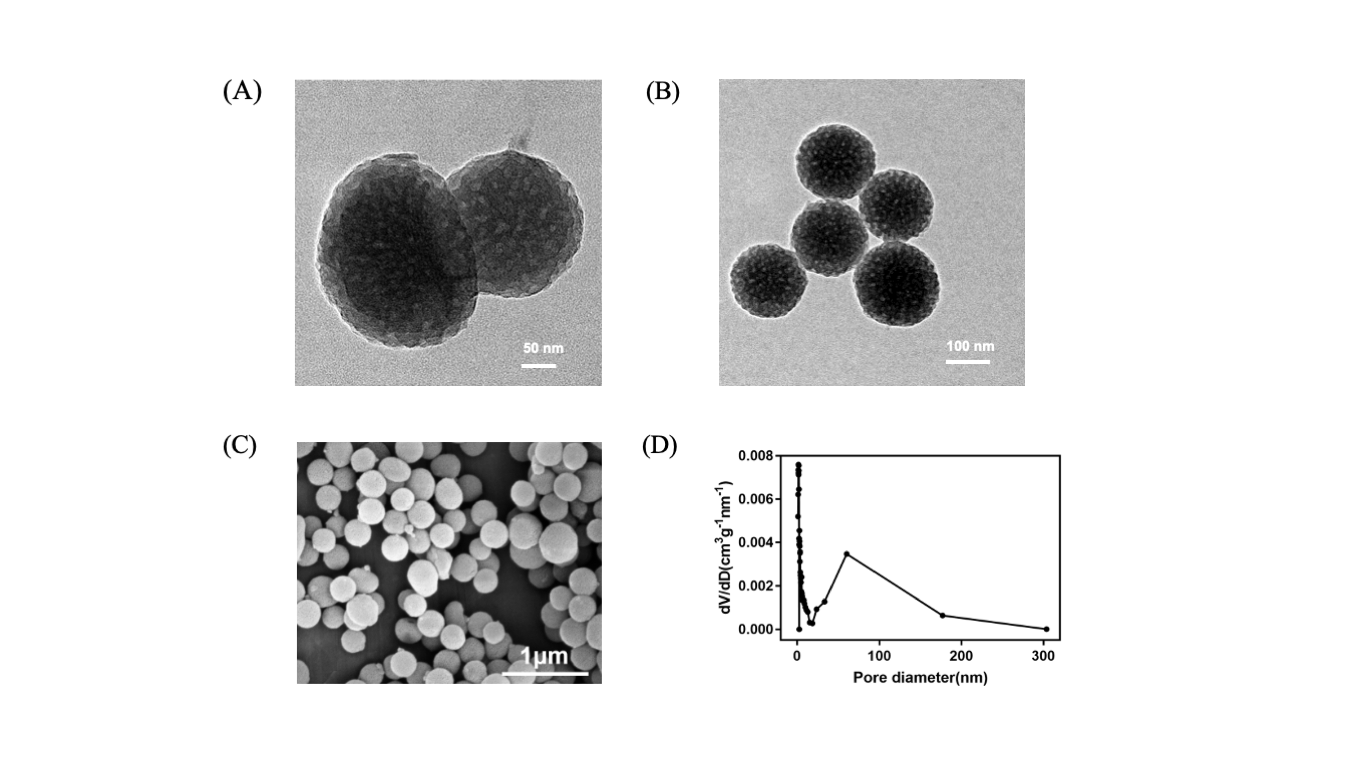


**Supplementary Figure 1.** (A-B) TEM images of MPDA NPs. (C) SEM image of MPDA NPs. (D) Pores diameters of MPDA NPs.

**
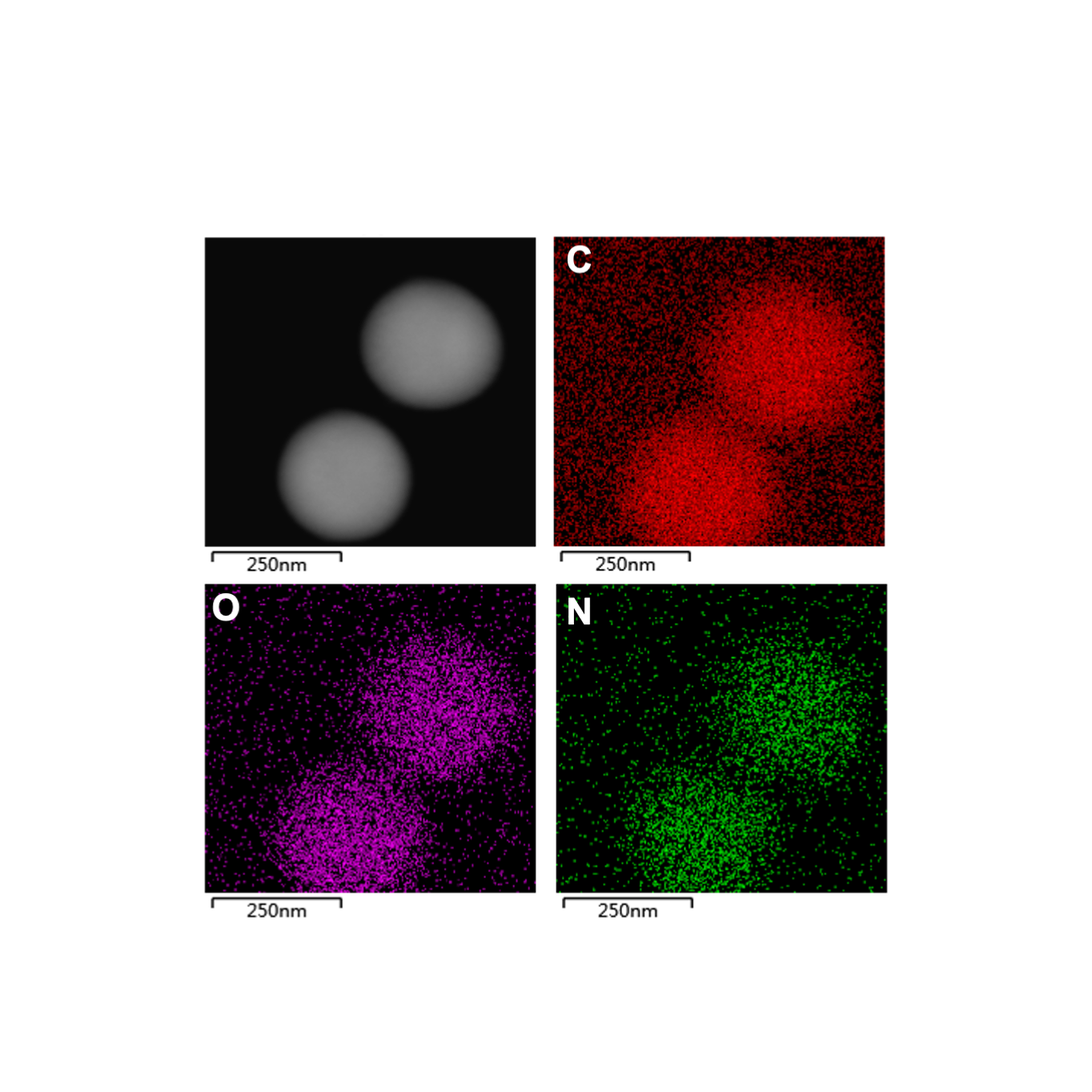
**

**Supplementary Figure 2.** Elemental mapping images of MPDA NPs.

**
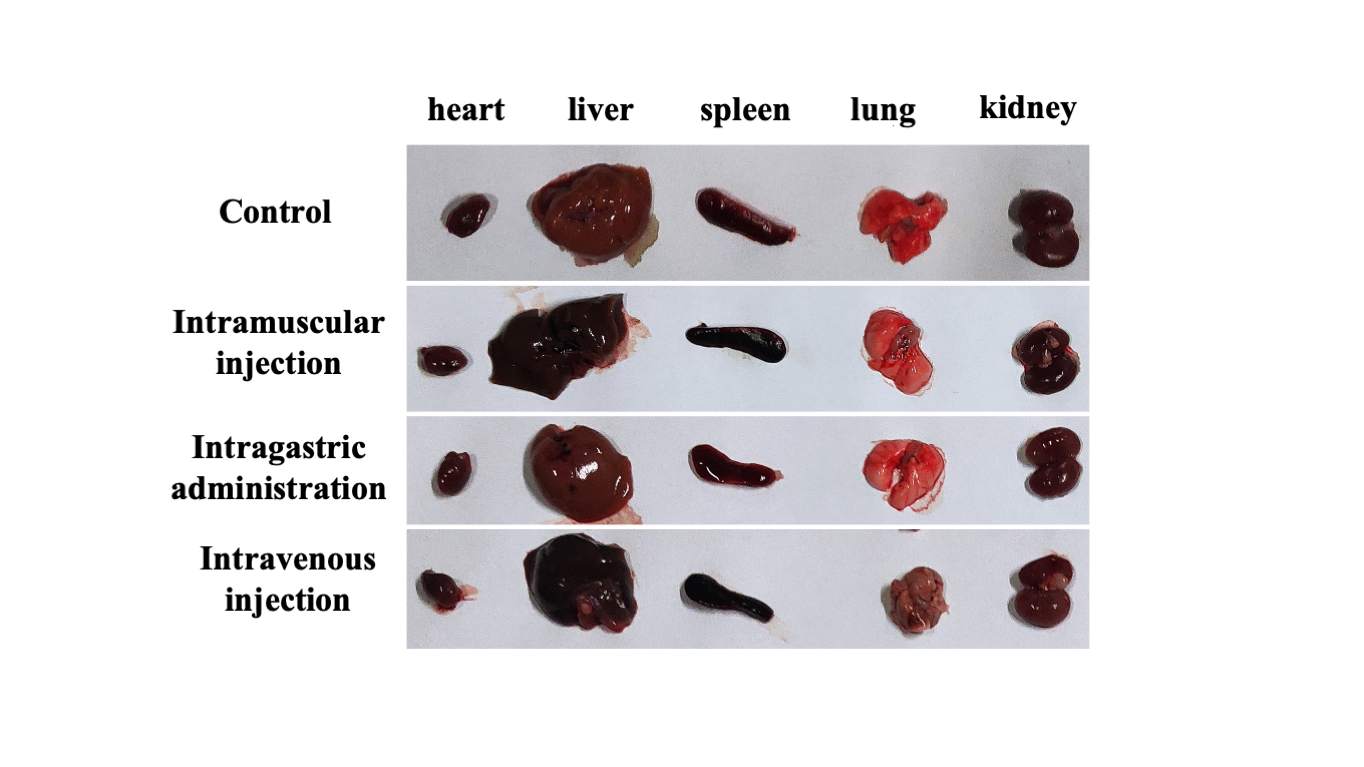
**

**Supplementary Figure 3.** Tissue appearance of tissues (heart, liver, spleen, lung and kidney) from the mice exposed by the intravenous injection, intragastric administration and intramuscular injection at day 7.


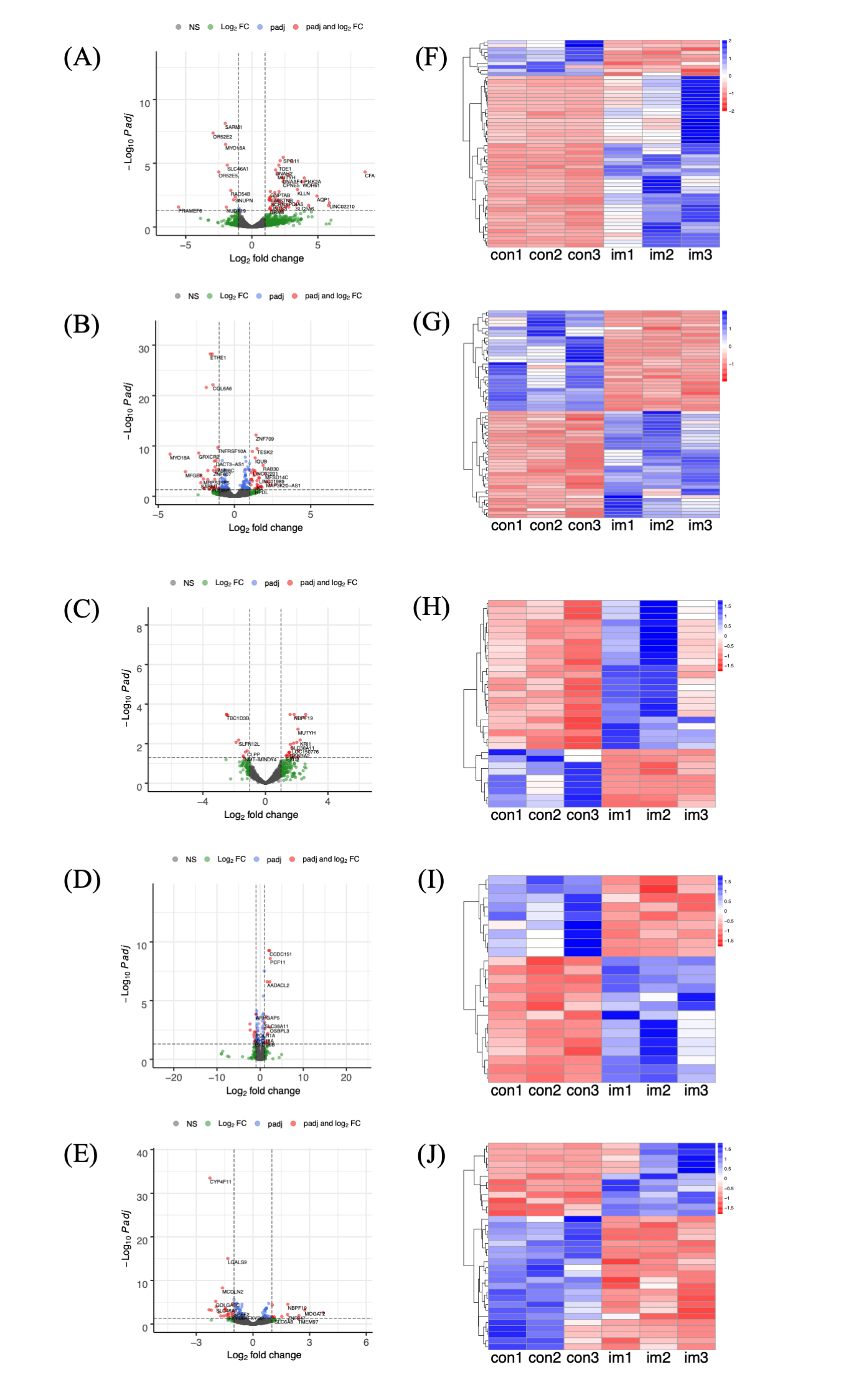


**Supplementary Figure 4.** (A-E) The volcano plots of DEGs between the intramuscular (i.m.) injection group and control group, for heart, liver, spleen, lung and kidney respectively. (F-J) The heatmap of DEGs.


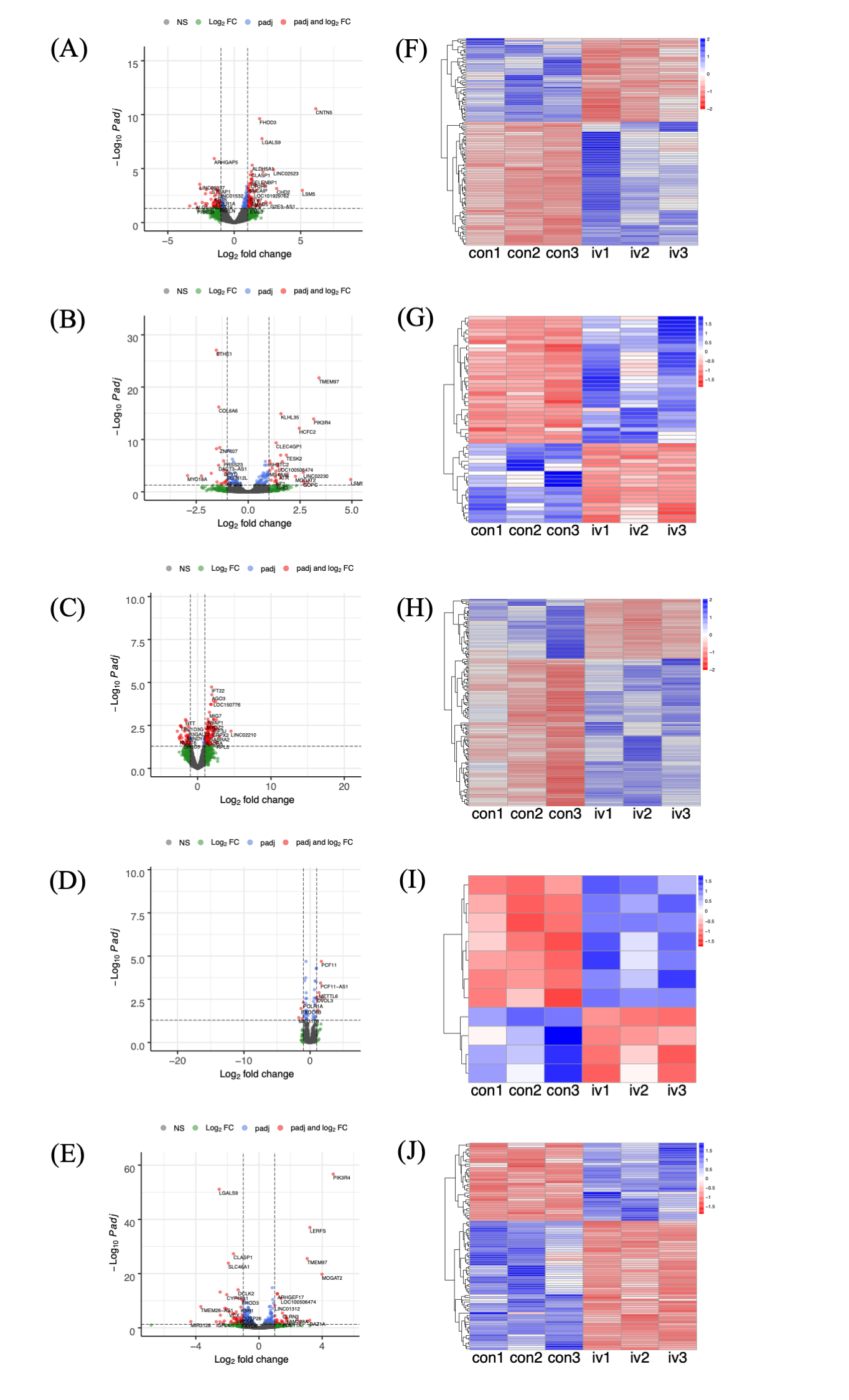


**Supplementary Figure 5.** The volcano plots of DEGs between the intravenous (i.v.) injection group and control group, for heart, liver, spleen, lung and kidney respectively. (F-J) The heatmap of DEGs.


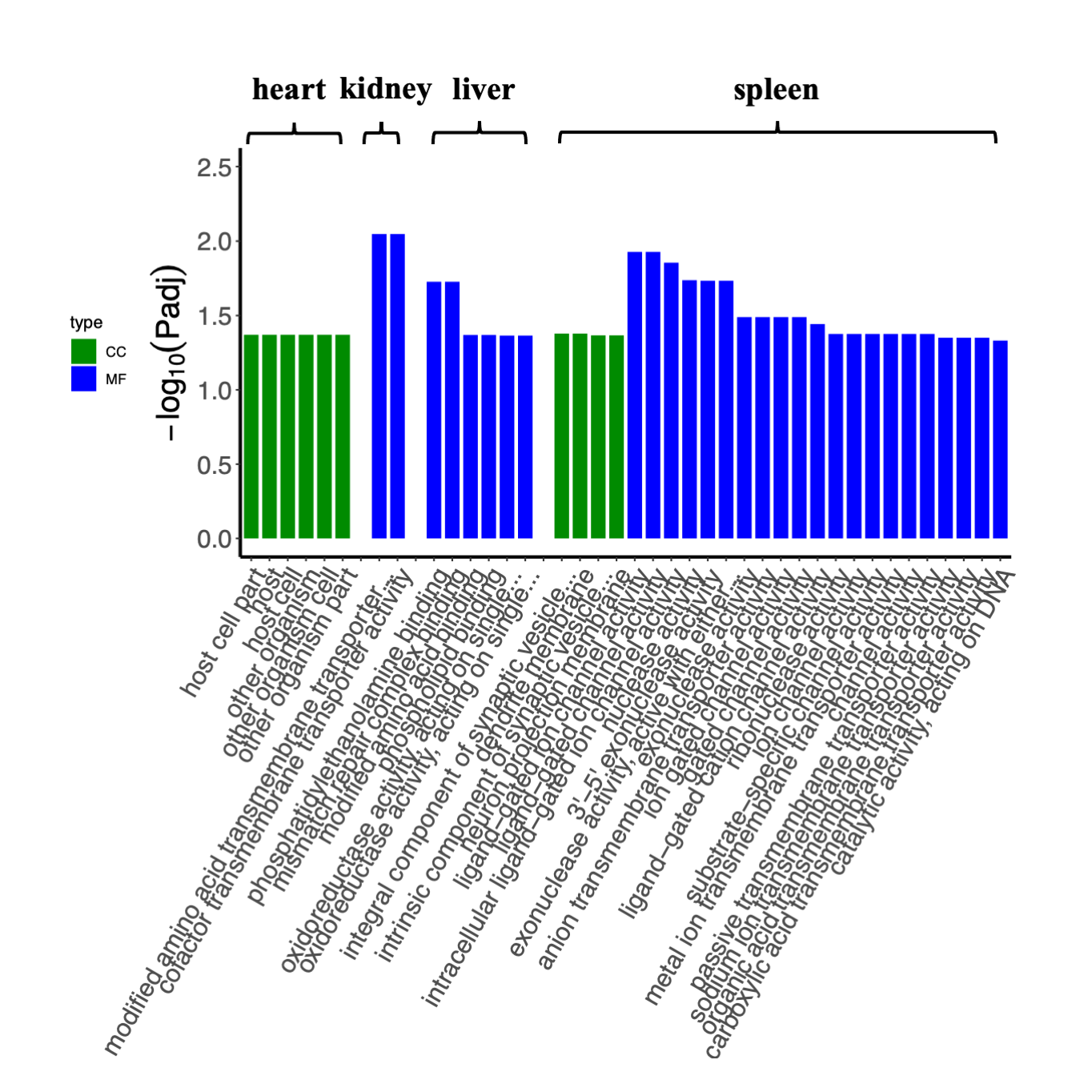


**Supplementary Figure 6.** The enriched GO terms of tissues (heart, kidney, liver, and spleen) from the intramuscular injection group. No GO term is enriched for lung.


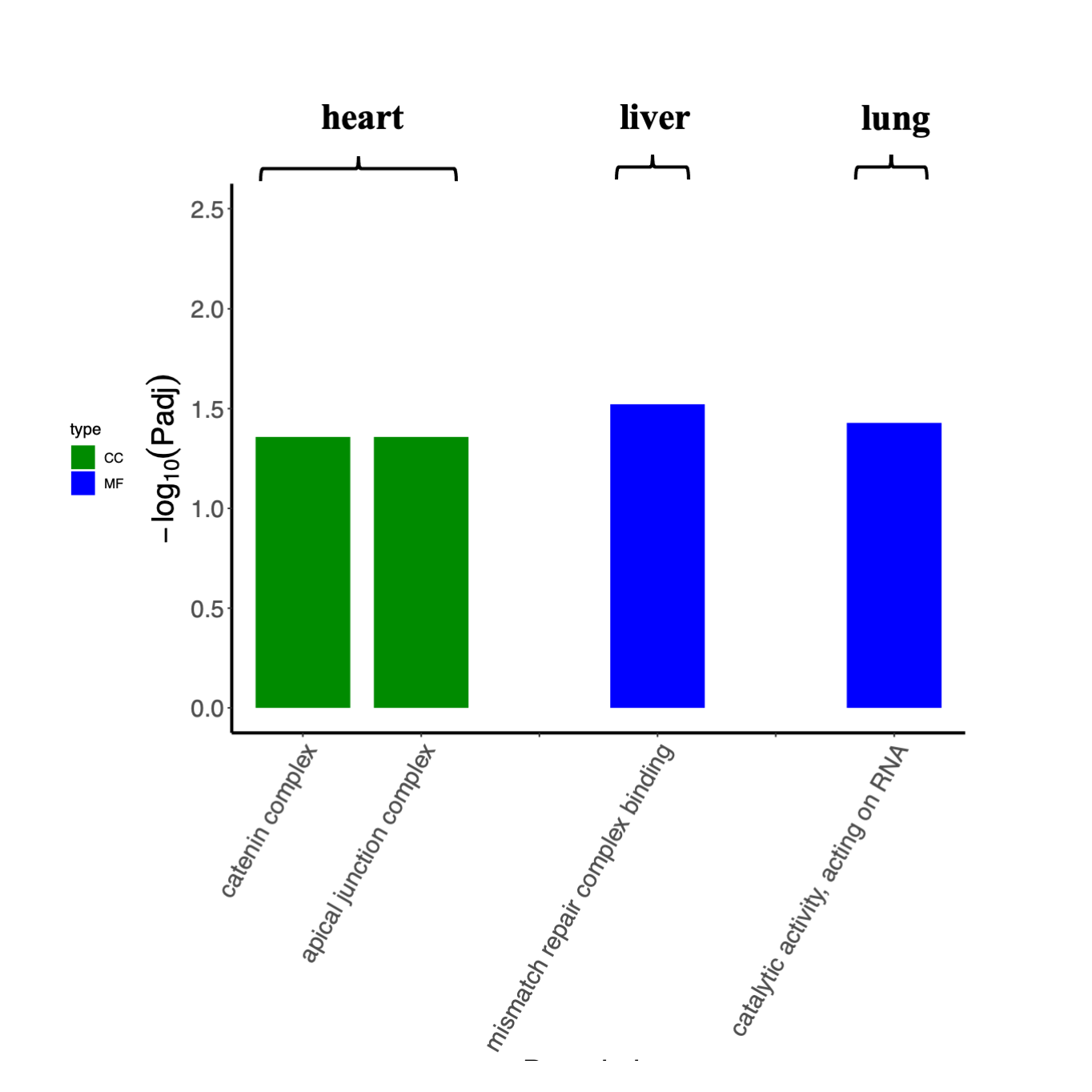


**Supplementary Figure 7.** The enriched GO terms of tissues (heart, liver, and lung) from the intravenous injection group. No GO term is enriched for spleen and kidney.


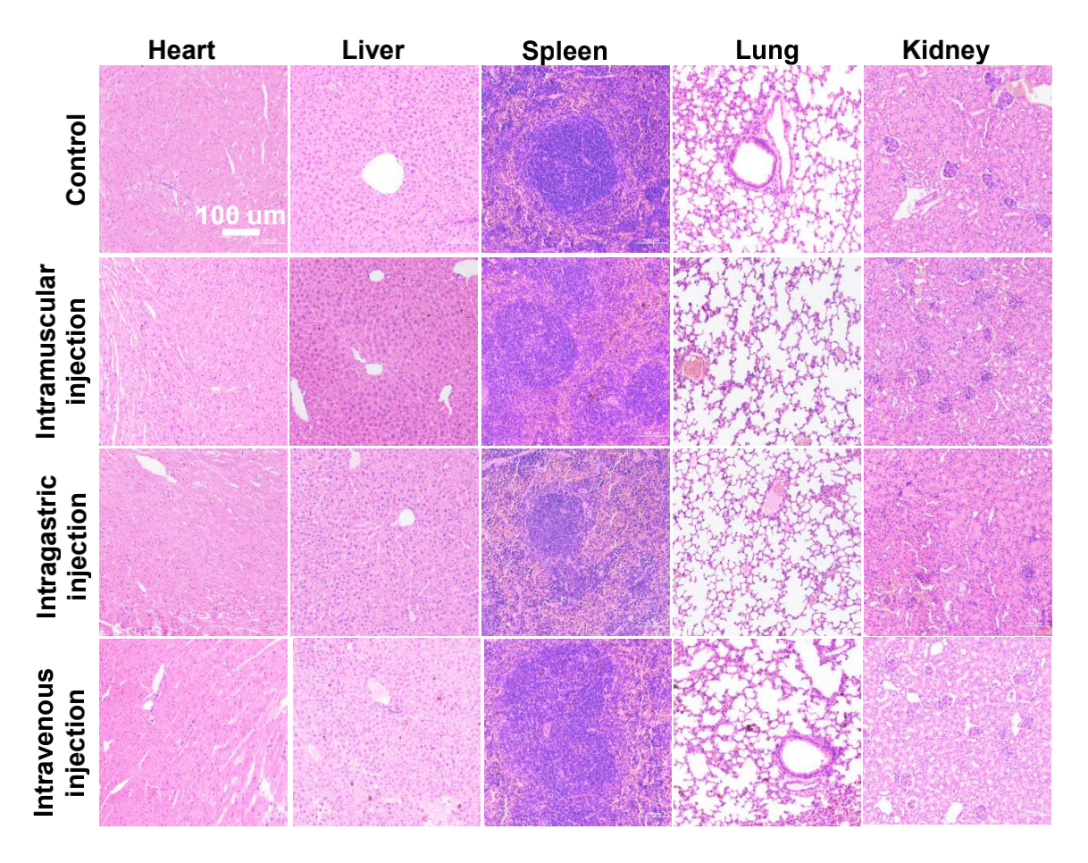


**Supplementary Figure 8.** Histological examination of tissues (heart, liver, spleen, lung and kidney) from the mice mice exposed by intramuscular (i.m.) injection, intragastric (i.g.) administration and intravenous (i.v.) injection at day 30. The scale bar is 100 μm.
